# Review of Alterations in Perlecan-Associated Vascular Risk Factors in Dementia

**DOI:** 10.3390/ijms21020679

**Published:** 2020-01-20

**Authors:** Amanda L. Trout, Ibolya Rutkai, Ifechukwude J. Biose, Gregory J. Bix

**Affiliations:** 1 Department of Neurology, University of Kentucky, Lexington, KY 40536, USA; A.trout@uky.edu; 2 Department of Neurosurgery, Clinical Neuroscience Research Center, Tulane University School of Medicine, New Orleans, LA 70112, USA; irutkai@tulane.edu (I.R.); ibiose@tulane.edu (I.J.B.); 3 Tulane Brain Institute, Tulane University, New Orleans, LA 70118, USA

**Keywords:** perlecan, extracellular matrix, basement membrane, VCID, vascular risk factors, and dementia

## Abstract

Perlecan is a heparan sulfate proteoglycan protein in the extracellular matrix that structurally and biochemically supports the cerebrovasculature by dynamically responding to changes in cerebral blood flow. These changes in perlecan expression seem to be contradictory, ranging from neuroprotective and angiogenic to thrombotic and linked to lipid retention. This review investigates perlecan’s influence on risk factors such as diabetes, hypertension, and amyloid that effect Vascular contributions to Cognitive Impairment and Dementia (VCID). VCID, a comorbidity with diverse etiology in sporadic Alzheimer’s disease (AD), is thought to be a major factor that drives the overall clinical burden of dementia. Accordingly, changes in perlecan expression and distribution in response to VCID appears to be injury, risk factor, location, sex, age, and perlecan domain dependent. While great effort has been made to understand the role of perlecan in VCID, additional studies are needed to increase our understanding of perlecan’s role in health and in cerebrovascular disease.

## 1. Extracellular Matrix and Perlecan

The basement membrane (BM) is a thin layer of extracellular matrices (ECMs) that anchors the epithelium (e.g., respiratory tract), mesothelium (e.g., peritoneal cavity), and endothelium (e.g., vasculature) to the underlying smooth muscle and connective tissue [1,2]. This specialized form of ECM is a scaffolding of interwoven macromolecules and proteins that provide structural support, contribute to differentiation, and serve as a signaling platform (reviewed in [3,4,5,6]). This dynamic interface represents a delicate balance among protein synthesis, proteolysis, and metabolism [4,7,8,9], contributing to cellular plasticity and tissue specificity. The vascular BM not only mediates compartmentalization but also creates a selective barrier to infiltrating proteins and cells in parts of the body, such as the brain, sustaining blood–brain barrier (BBB) integrity [4,7,8,9]. Ranging from 20 to 200 nm, the ECM consists of over 20 proteins, including a number of glycoproteins such as laminins, collagen IV, nidogens, and heparan sulfate proteoglycans (HSPG) (reviewed in [3,4,5,6]).

Perlecan is one of the most abundant heparan sulfate proteoglycans (HSPG) [10], encoded by *HSPG2* on chromosome 1 [11,12,13]. The role of perlecan has been studied in a multitude of cellular processes ranging from cell adhesion [14,15], wound healing [16], angiogenesis [17,18,19,20], neuroprotection [18,21], and normal development of the heart, bone, cartilage, and brain [22,23,24,25,26,27]. Perlecan consists of a core protein with a size between 467 kDa (humans) and over 750 kDa with the addition of three to four glycosaminoglycan (GAG) side chains [28,29]. This multi-domain molecule is composed of five distinct regions, termed domains one to five (DI–V) (reviewed in [30,31,32]). DI contains a Sperm, Enterokinase and Agrin fold with GAG and heparan sulfate (HS) attachment sites that has been proposed to facilitate the release of heparan binding growth factors in wound healing [33,34,35]. This domain has a distinct, perlecan specific protein motif that does not share homology with any other proteins [36]. Truncated perlecan DI, found in *Hspg2*^−/−^ or perlecan knock out mice, has been associated with complete loss of function, embryonic lethality (~E10–12), enlarged ventricles, smaller brains, and weakened vasculature leading to severe bleeding and heart malformations [24,25,37]. Rescuing this phenotype via cartilage-specific perlecan (*Hspg2^−/−^; Col2a1-Hspg2^TG/−^*) knock in [38] results in 50% more survivability at ~E10–12 [39], normal cephalic development [39], intact BBB [40], but decreased neurogenesis [41], and endothelial [42] and pericyte [40] dysfunction following injury. Interestingly, an altered attachment site of DI for HS side chains, due to the lack of exon 3 (*Hspg2*^∆3/∆3^), did not affect perlecan expression [43], nor associated with birth defects or phenotypic developmental abnormalities other than congenital cataracts [43]. However, vascular injury, induced by the ligation of the distal carotid artery in the *Hspg2*^∆3/∆3^ mice has been found to contribute to an increased intimal hyperplasia [44]. Moreover, smooth muscle cells, isolated from *Hspg2*^∆3/∆3^ have shown altered binding of fibroblast growth factor (FGF-2) and a greater proliferation capacity when compared with wild type [43]. DII, containing four low density lipoprotein (LDL) receptor motifs and an immunoglobulin-like (IG) fold, is suggested to play a role in development via wingless (Wnt), LDL, and calcium signaling [45,46,47,48], whereas DIII contains laminin epidermal growth factor (EGF) and laminin IV type A (laminin B) domains that can directly bind fibroblast growth factor [48,49,50]. Perlecan-deficient endothelial cells, via antisense targeted against DIII, have been associated with an increased thrombotic occlusion rate, possibly due to the inability to bind FGF [51]. A C1532Yneo mutation in DIII in perlecan hypomorph (pln^−/−^) mice results in more than 90% reduction in perlecan expression [52]. These mice display musculoskeletal tissue disorders, resembling the Schwartz–Jampel syndrome (SJS) in humans [52], a disease due to *HSPG2* gene mutation [53,54,55,56,57,58,59,60]. Furthermore, larger infarcts and worse functional deficits have been reported in perlecan hypomorph mice following middle cerebral artery occlusion (MCAo) [18,61]. The repeating Ig C2-type modules in DIV, with or without additional GAG attachment site, determine the adhesion properties of perlecan to other ECM proteins [47,62]. The perlecan C-terminal, termed endorepellin or DV, contains three laminin G-like subdomains with dual EGF-like domains [36,48] and can be cleaved by proteases such as matrix metalloproteases (MMPs) or cathepsins [18,61,63,64,65,66,67]. DV has been linked to anti-angiogenic activity in tumor growth [20], pro-angiogenic and neuroprotective effects in ischemia [19] as well as to amyloid beta (Aβ) toxicity [17,21,68]. Interestingly, the third laminin G-like subdomain (LG3) appears to be particularly bioactive and may convey much of DV’s reported biological activity [18,20].

Overall, these observations provide strong evidence that perlecan is essential for brain, bone, heart, and cartilage development and plays a critical role in the maintenance of homeostatic balance in the brain following injury.

## 2. Perlecan and the Cerebrovasculature in Disease and Stroke

The cerebral vasculature is composed of distinct types of vessels, i.e., pial arteries, arterioles, capillaries, or venules that are structurally and biochemically different, contributing to their specificity. The pial arteries consist of three main layers from inside out: the tunica intima that contains both endothelial cells and BM, surrounded by the tunica media, compromised by smooth muscle cells and the most outer part, the tunica adventitia (or connective tissue). As these vessels dive into the brain parenchyma and branch into smaller and smaller segments, they give rise to brain arteriole and capillary networks, while losing their smooth muscle (SMC) coverage but gaining more pericytes and astrocytic endfeet. All of these cells regulate and/or secrete perlecan [15,40,69,70,71,72,73,74,75,76]. Studies investigating the effects of altered perlecan expression are contradictory, ranging from pro vs. anti-angiogenic [17,18,70,77], or associated or not with plaque and thrombotic core proteins [78,79,80,81,82,83,84]. The role of perlecan may depend on the model, size of the blood vessel, sex (female vs. male), and age.

Cerebrovascular diseases represent one of the top five most common causes of death in the United States [85]. As we age, our vasculature undergoes degeneration due to the accumulation of mechanical and sheer stress induced by innate fluctuations in blood pressure [86]. Arteriolosclerosis, arterial stiffness, and reduced compliance, are among the first pathologies to present, leading to altered cerebrovascular blood flow (CBF) and ECM protein metabolism, or the remodeling of ECM. Moreover, proteases known to cleave perlecan such as matrix MMPs or cathepsins [18,61,63,64,65,66,67] increase in cerebrovascular diseases (reviewed in [87]). Fluctuation of perlecan expression has been reported in aging; perlecan levels in mouse brain were high at 3 months, decreased at 8 months, followed by a secondary increase at 16 months of age [88]. However, a study by Kerever et al. reported no change in perlecan expression in the subventricular zone of aged mice without comorbidity [89]. Age-related structural changes are associated with decreased CBF, contributing to cell senescence, damage, and dementia [90,91,92,93,94,95,96,97,98]. A term that encompasses cognitive decline associated with vascular change is Vascular contributions to Cognitive Impairment and Dementia or VCID. VCID is the second leading cause of dementia [99,100,101] behind Alzheimer’s disease (AD) [102,103,104,105] with many of the same vascular risk factors, such as, age, sex, hypertension [106], atherosclerosis, and diabetes mellitus (DM) [107,108,109,110,111,112,113,114]. How metabolic changes such as high glucose, high insulin, or high free fatty acid levels individually or in combination affect perlecan distribution and expression is reviewed in Table 1. VCID etiology ranges from cerebrovascular disruption, seen in small vessel diseases of the brain, including arteriolosclerosis, and cerebral amyloid angiopathy (CAA, pathologic accumulation of amyloid beta (Aβ) protein in brain blood vessels), to the profound symptomatic damage following acute stroke. These vascular risk factors may increase BM thickness contributing to the narrowing of vascular lumen and to the disruption of BBB integrity. Changes in the BBB are dynamic, as it opens and closes multiple times within the first hours to days after ischemic injury [115]. This disrupted BBB provides a pathway for inflammatory cells that may infiltrate the brain and release additional proteases, free radicals, chemokines, and cytokines [116,117,118]; further contributing to altered ECM synthesis of matrix proteins or proteolysis, splice variants, smooth muscle cell migration and proliferation [119]. The extent of the injury and remodeling in the ECM is determined by the vascular injury, loss of blood flow, resultant inflammation, and ultimately the changes in oxygen and nutrient supply to the neurons and their support cells (glia and immune cells).

Atherosclerosis, a common cause and comorbidity in stroke and vascular dementia, has been linked with the distribution of ECM proteins (e.g., perlecan) in the walls of peripheral arteries. Atherosclerosis, a disease of large and medium sized arteries, develops in stages, starting with intimal hyperplasia due to lipid accumulation, followed by development of plaques and thrombosis leading to vessel occlusion (ischemic stroke) or rupture (hemorrhagic stroke) [135,136,137,138]. Perlecan has been linked to lipid retention in the vasculature [83], possibly through the LDL receptor motifs region in DII (additional studies on perlecan and atherosclerotic plaques are outlined in Table 2). Increased BM perlecan expression has been correlated with atherosclerotic lesions in experimental mice [79,81], non-human primates [139], and in humans [80]. Interestingly, perlecan expression was found to be increased in aged human cerebral arteries but decreased as the lesion progressed [140]. Haung et al. have shown that perlecan accumulation via angII-driven mechanisms preceded the progression of atherosclerosis [81]. This suggests that perlecan accumulation may increase endothelial barrier disruption resulting in lesion formation via increased deposition of LDL. Moreover, the loss of perlecan has been associated with less atherosclerosis in early lesions of mice without effect on the lipoprotein profile [83,141].

Also, decreased heparan sulfate and perlecan levels were detected in fully developed atherosclerotic lesions of tunica intima, derived from non-diabetic and Type 2 non-insulin-dependent diabetes mellitus patients [142], as well as from those with carotid stenosis [143], respectively. Hence, it is plausible that the distribution of various ECM components, especially perlecan, drives the initiation and progression of atherosclerosis. In contrast, an earlier study by Hollmann et al. reported decreased perlecan expression, associated with advancing age and severity of atherosclerosis, in both normal and atherosclerotic regions of human aorta [80]. The difference between this study and others may lie in the method of tissue preparation and perlecan detection. Additional contradictory studies show perlecan preventing atherosclerosis [84,144,145]. In an in vitro model, increased endothelial cell HSPG/perlecan production led to a reduction of the atherosclerotic lesions [146].

Amyloid that accumulates within the vasculature, CAA, and brain parenchyma, forms plaques (AD) that disrupt the distribution of perlecan (Table 3) [147,148,149], decreases the ability of SMC to bind perlecan [150], and contributes to vascular wall weakening [147]. Modifications to the sulfate content of the specific GAG chains of perlecan were also found to be critical to the binding of amyloid [151]. In a systematic family-base genome-wide association and meta-analysis that included 15 million imputed variants from 3524 European subjects, HSPG variant rs2445130-A (p = 8 × 10^−7^) had an increased association of AD status and onset age [152]. Furthermore, HSPGs have been found in amyloid deposits in congophilic angiopathy as well as in neuritic plaques [153]. Similarly, Lepelletier et al. reported an association in amyloid regions with high perlecan in patients with a Braak stage >2 [154] when using antibodies targeting DIV. However, a study by Verbeek et al. found agrin, and a lesser extent glypican and syndecan, to be the major HSPGs associated with amyloid, whereas perlecan was not found in hippocampal nor neocortical plaques when using antibodies against DI-DII and DIV [155]. The lack of perlecan staining on amyloid positive vessels of CAA patients has been reported as well [156]. These observations suggest the possibility that the pathologic absence of perlecan/DV contributes to, and/or is the result of cerebrovascular Aβ accumulation. Functionally, treatment with a recombinant DV of perlecan has been demonstrated to block the deleterious effects of amyloid on brain endothelial cells [17] via α5β1 integrin receptor, a key angiogenesis receptor, downregulated after brain development, and only to be upregulated in response to ischemic brain injury [157]. The role of perlecan in amyloid accumulation and clearance may be intricately regulated by cell types, vary among species, and perlecan domain dependent.

Stroke results from the disruption of blood flow to the brain due to the narrowing or occlusion of a vessel (ischemic) or to the weakening of the vessel wall (hemorrhagic). Perlecan was found to be lower in non-human primate models of focal cerebral ischemia [63,169], whereas DV has been demonstrated to increase in rodent models of stroke (endothelin-1 and tandem common carotid artery occlusion and distal middle cerebral artery occlusion) [18]. The therapeutic potential of recombinant DV after ischemic stroke has been demonstrated by us and others [18,40]. Correspondingly, the lack of perlecan, when using hypomorphs, *Col2a1-Hspg2^TG/−^* and *Hspg2^−/−^* mice, was associated with larger infarcts as well as worse functional outcomes after MCAo and decreased neurogenesis, respectively [18,40]. Increased perlecan expression was found following traumatic brain injury in mice [75] as well as in human samples from patients with brain arteriovenous malformation [170]. Similarly, Nugent el al. showed increased thrombotic occlusion rates in perlecan-deficient endothelial cells in a porcine carotid artery injury model [51], not surprisingly as perlecan antithrombotic/anticoagulant studies have shown perlecan directly binding to anti-thrombin III [171].

Hypertension is an important risk factor for stroke and vascular dementia and can occur at any age as well as in pregnancy. Vascular remodeling of peripheral and cerebral blood vessels is a common feature of hypertension that has been linked to changes in perlecan (Table 4). Various studies have shown that increased expression and accumulation of collagen IV and elastin in peripheral and cerebral arteries lead to arterial stiffness. However, only a small number of investigators have studied the expression of perlecan in various models of hypertension. McGuire and colleagues were the first to report increased expression of perlecan in the aorta of spontaneously hypertensive rats (SHR), a model of essential hypertension [172]. This finding was corroborated in the aorta of hypertensive rats, induced by high salt diet [173]. Nonaka et al. reported reduced endothelium-dependent relaxation through lower endothelial nitric oxide synthase (eNOS) expression in aortas of perlecan-deficient mice and when perlecan is knocked-down in human aortic endothelial cells [42], suggesting that perlecan may play a role in vasodilation. Furthermore, angII and Western diet have been reported to increase level of perlecan in biglycan deficient mice [82]. Haung et al. [81] showed that perlecan accumulation, via angII-mediated mechanisms, precedes the progression of atherosclerosis, suggesting that perlecan accumulation may facilitate endothelial barrier disruption, resulting in an increased deposition of LDL, the hallmark of atherosclerotic lesion formation. However, no change was detected in perlecan expression in a coarctation of abdominal aorta model of hypertension in rats [174]. In pregnant women, an association was found between increased plasma concentration of perlecan and early onset of gestational hypertension [175]. The authors attributed their findings to impaired microvascular perfusion leading to the degradation of glycocalyx of the kidneys. Likewise, early preeclampsia with or without HELLP (hemolysis, elevated liver enzymes, and a low platelet count) syndrome was associated with an increased placental perlecan expression that showed a positive correlation with maternal vascular malperfusion [176]. In contrast, the findings by Guo et al. showed decreased perlecan concentration in urine when compared the gestational hypertension group with controls, indicating glomerular filtration barrier injury and renal dysfunction [177]. Collectively, these observations paint a complex and underexplored role for perlecan in hypertension in the cerebrovasculature and beyond.

## 3. Conclusions

Besides aging, atherosclerosis, hypertension, and diabetes are cerebrovascular risk factors for VCID, dementia and stroke, making it difficult to tease out the underlying mechanisms of cognitive decline. In addition, changes in perlecan expression and distribution in response to VCID appears to be injury, risk factor, location, sex, age, and perlecan domain dependent. Overall, great effort has been made in recent decades in understanding the role of perlecan in VCID. While recombinant perlecan DV, in mice and rats, has been found to be angiogenic, neuroprotective, and neuroreparative [17,18,19,68,157,178,179,180], new studies are warranted to address the role of perlecan and its domains in health and in combination with cerebrovascular risk factors.

## Figures and Tables

**Table 1:** Metabolic Changes and Perlecan.

Model [Organism], Age	Tissue/Sample	Sex	Findings: Changes in Perlecan (Domain, Antibody; Where Applicable)	Reference
STZ and osteoarthritis (ACLT) [WKY rats], 5–6 mo	Condylar femur, tibial articular cartilage	Male	↓ mRNA and protein with hyperglycemia ↑ mRNA with hyperglycemia and osteoarthritis	[120]
db/db and db/+ non-diabetic [mice], 10–12 wk	Kidney	Mixed	− (no change) in perlecan (DIV, clone A7L6-MAB1948)	[121]
STZ-DN [C57BL/6 mice], 6–8 wk	Kidney	Male	↓ perlecan core protein, no change in mRNA stability in DN vs. control	[122]
STZ [SD rats], 5 and 12 mo	Kidney	Male	− (no change) in perlecan (DI, clone16)	[123]
STZ [C57Bl/6 mice], 6 mo	Liver	Male	↓ perlecan	[124]
DN [Human]	n. sp.	n. sp.	Association between *HSPG2* variant and DN	[125]
Focal Segmental Glomerulosclerosis and DN [Human; CD44^+/+^ and CD44^−/−^ and 24 wk BTBR ob/ob mice]	Kidney	Male (mice)	↑ perlecan (DIV, clone A7L6)	[126]
DN [Human]	Kidney	n. sp.	↑ perlecan (DIV, clone A7L6)	[127]
Non-ischemic kidney injury, db/db (BKS.Cg-m+/+Lepr^db,^ diabetic) and db/m non-diabetic [mice], 20 wk	Kidney	Male	↓ Glomerular level in diabetic vs. non-diabetic	[128]
IDDM/DN ± albuminuria [Human]	Genomic DNA from leukocytes	n. sp.	Association of a *Bam*Hl HSPG2 polymorphism in DI with diabetic nephropathy	[129]
IDDM/NIDDM ± diabetic retinopathy	Eye	n. sp.	− (no change)	[130]
High glucose, inflammation, Short treatment [Human]	Human umbilical cord vein endothelial cells	n. sp.	↑ perlecan following IL-1β treatment ↑ HS chains by TNF	[131]
Wound healing [Retired breeder Lewis rats and Zucker Diabetic Fatty diabetic rats]	Skin	Male	↑ perlecan (DI, clone CCN-1) in healing and blood vessel formation with chitosan scaffolds	[35]
DN, non-diabetic [Human], mean age of 64 yr and 39.3 yr	Parietal epithelial cells	Mixed	↑ TGF, advanced glycation, and high glucose	[132]
High glucose [Human]	Trophoblast cell line 3A-Sub-E	n. sp.	↓ perlecan (DIV, clone A7L6)	[133]
[Human] gestational age 5–7wk, 15–26 wk, 36–40 wk	Placenta	n. sp.	↓ (DIV, clone A7L6) during placental maturation in normal pregnancies ↑ in gestational diabetes	[134]

Abbreviations. ↑: increase; ↓: decrease; −: no change; ACLT: anterior cruciate ligament transection; BTBR ob/ob: black and tan, brachyuric, obese (leptin-deficiency); DI: domainI; DIV: domain IV; db: diabetic (leptin defiency); DN: diabetic nephropathy; HS: Heparan sulphate; *HSPG2*: Heparan sulphate proteoglycan 2; IDDM: insulin-dependent diabetes mellitus; mo: month; NIDDM: non-insulin-dependent diabetes mellitus; n. sp.: not specified; SD: Sprague–Dawley; STZ: streptozotocin; TGF: transforming growth factor; TNF: tumor necrosis factor; wk: week; WKY: Wistar Kyoto; yr: year.

**Table 2:** Atherosclerosis and Perlecan.

Model [Organism], Age	Tissue/Sample	Sex	Findings: Changes in Perlecan (Domain, Antibody; Where Applicable)	Reference
ApoE and LDLR KOs [mice]	Aorta	Mixed	↑ perlecan in intima and smooth muscle cells	[79]
[Human]	Aorta	Male	↑ perlecan (DIV, clone A7L6, RT-794-B1) with age and lesion progression	[80]
LDLR−/− and AT1a−/− LDLR−/− [mice]	Aorta	Female	↑ perlecan with lesion	[81]
AngII and Western diet [Biglycan deficient/WT mice]	Aorta, carotid artery	Mixed	↑ perlecan with Western diet in biglycan deficient mice not WT − No change in perlecan by angII	[82]
ApoE0/*Hspg2^Δ3/Δ3^*, ApoE0, and C57BL/6 [mice], 15 and 33 wk	Aorta	Mixed	↓ perlecan (core protein, R14) at 15 and 33 wk with smaller lesion − No change in perlecan mRNA	[83]
Normal or Paigen diet [Heterozygous perlecan-deficient and apoE0 mice], 12, 20 and 24 wk	Aorta	Mixed	− No change in lipid profile upon perlecan deletion ↓ atherosclerosis in young heterozygous KO/apoE0 ↑ perlecan staining in subendothelial region with atherosclerosis	[141]
[Human] 32–88 yr	Atherosclerotic carotid plaques, control iliac, mesenteric arteries, and aorta	Mixed	↓ perlecan (R14) protein and mRNA in atherosclerotic group	[84]

Abbreviations. ↑: increase; ↓: decrease; −: no change; AngII: Angiotensin II; ApoE: apolipoprotein E; ApoE0: apoE gene knockout; AT1a: angiotensin II subtype-1a; DIV: domain IV; *Hspg2^Δ3/Δ3^*: Heparan sulphate proteoglycan 2 with deleted exon 3; KO: knock out; LDLR: low density lipoprotein receptor; wk: week; WT: wild type; yr: year.

**Table 3:** Amyloid and Perlecan.

Model [Organism], Age	Tissue/Sample	Sex	Findings: Changes in Perlecan (Domain, Antibody; Where Applicable)	Reference
Subclinical and clinical AD, Controls [Human]	Post-mortem Cortex, frontal and temperoral superior frontal gyrus and inferior temporal gyrus	Mixed	↑ in perlecan (DIV, 2501) in brain regions that have ↑ Aβ assoc. with Braak >2. Similar expression in subclinical and clinical. Not associated with vascular density	[154]
11 DAT patients [Human]	Frontal and temporal neocortex and hippocampus	Mixed	Perlecan (DIV, MAB 1948) and DI-DIIa, MAB95J10) not associated with senile plaques and tangles	[155]
LDLR and apoE mice	Proximal aortic tissue	Mixed	↑ in perlecan (EY9) associated with serum amyloid A	[78]
Aβ stereotaxic injections TRE4, and C57Bl/6, 16 mo, [mice]	Capillaries	Male	− (no change) in perlecan	[158]
TBI/CCI [rat], Juvenile (17 day old)	Ipsilateral cortex	Male	↑ in perlecan (DV, H300), which coincides to increased amyloid expression − (no change) via WB	[159]
SAMP8 [mice], 6 mo	Hippocampus	Male	− (no change) perlecan and it is not associated with amyloid granules	[160]
APP/PS1 Amyloid transgenic and c57Bl/6J [mice], 12 mo	Cerebral tissue	Male	↑ in perlecan associated with amyloid plaque and Apo B lipoprotein	[161]
Tg2576 and C57BL6 [mice], 3, 7, 22 mo	Hippocampal capillaries and arteries	Mixed	perlecan ↑ 3 m, ↓ 8 m, and ↑ 16 m in capillaries	[88]
C57BL/6 [mice],	Cortical and hippocampal neurons	Mixed	↑ in perlecan DV and LG3 resulted in ↓ Aβ neurotoxicity	[21]
C57BL/6 [mice]	Cerebrovascular endothelial cell line	n. sp.	↑ DV rescued the Aβ decreased proliferation	[17]
C57BL/6 [mice] and [Human]	Cortical neurons	n. sp.	↑ in perlecan DV resulted in ↓ Aβ neurotoxicity	[68]
APPswe/PS1dE9 [mice]	Vascular	Female	↓ perlecan (DIV, MAB1948) in Aβ-positive vessels	[162]
LOAD Chinese [human]	Genomic DNA from peripheral venous blood leukocytes	Mixed	− No association with LOAD and *HSPG2* polymorphism (*HSPG2* gene, rs 3767140 C/A)	[143]
LOAD Finnish [human]	Genomic DNA from peripheral venous blood leukocytes	Mixed	↑ association with LOAD and *HSPG2* polymorphism in APOE carriers	[163]
LOAD Jewish [human]	Genomic DNA from peripheral venous blood leukocytes	Mixed	− No association with LOAD and *HSPG2* polymorphism (*HSPG2* gene, rs 3767140 C/A)	[164]
FBD, FDD, and AD [human]	Hippocampal formation with temporal cortex and white matter, frontal cortex	n. sp.	↓ perlecan (DIV, 1948) in FBD CAA and plaques and FDD CAA − No association perlecan in FDB and FDD diffuse and all sporadic and variant AD (CAA plaque, and diffuse)	[165]
LPS verse control treated [*Mustela Vison*-mink]	Spleen	n. sp.	↑ in perlecan (DIV, 1948) associated amyloid	[166]
A/J mice	Liver	Mixed	↓perlecan associate with ↓ amyloid	[167]
[human]	Cerebellum and cerebrum	Mixed	↓ perlecan (DI and DII, 95J10 and DIV, 1948) in senile plaques (non-fibrillar and fibrillar)	[168]
AD and CAA (Dutch) [humans]	Frontal, temporal, parietal, and occipital neocortex	Mixed	↓ perlecan (DI and DII, 95J10, and DIV, 1948) in CAA and in senile plaques	[156]

Abbreviations. ↑: increase; ↓: decrease; −: no change; AD: Alzheimer’s Disease; Aβ: amyloid beta; A/J: Apoa2^C^ allele; APP: amyloid precursor protein (swe: Swedish mutation); PS1: presenilin1 (dE9 mutation); CAA: cerebral amyloid accumulation, CCI: control cortical impact, DAT: dementia of the Alzheimer type; DI; domain I; DII: domain II; DIV: domain IV; DV: domain V; FBD: familial British dementia; FDD: familial Danish dementia; *HSPG2*: Heparan sulphate proteoglycan 2; LDLR: low density lipoprotein receptor; mo: month; n. sp.: not specified; SAMP8: senescence accelerated mouse 8; LOAD: late onset Alzheimer’s Disease; LPS: lipopolysaccharide; TBI: traumatic brain injury; WB: western blot; wk: week; yr: year.

**Table 4:** Hypertension and Perlecan.

Model [Organism]	Tissue/Sample	Sex	Findings	Reference
Preeclampsia [Human]	Urine	Female	↓ perlecan compared to normotensive controls	[177]
SHRSP [Rat]	Aorta	Male	↑ perlecan	[172]
Coarciation [Sprague–Dawley Rats]	Aorta	Male	− (no change) in perlecan	[174]
±HELLP syndrome [Human] gestational age 28.4–39.7 wk	Placenta	n. sp.	↓ perlecan (MP4) during placental maturation ↑ perlecan in early pregnancy loss and in preeclampsia without HELLP ↓ perlecan in late preeclampsia	[176]

Abbreviations. ↑: increase; ↓: decrease; −: no change; HELLP: hemolysis, elevated liver enzymes, and a low platelet count; n. sp.: not specified; SHRSP: spontaneously hypertensive stroke prone rats; wk: week.
